# 180,000 Years of Climate Change in Europe: Avifaunal Responses and Vegetation Implications

**DOI:** 10.1371/journal.pone.0094021

**Published:** 2014-04-09

**Authors:** Sandra Ravnsbæk Holm, Jens-Christian Svenning

**Affiliations:** Ecoinformatics and Biodiversity, Department of Bioscience, Aarhus University, Aarhus C, Denmark; University of York, United Kingdom

## Abstract

Providing an underutilized source of information for paleoenvironmental reconstructions, birds are rarely used to infer paleoenvironments despite their well-known ecology and extensive Quaternary fossil record. Here, we use the avian fossil record to investigate how Western Palearctic bird assemblages and species ranges have changed across the latter part of the Pleistocene, with focus on the links to climate and the implications for vegetation structure. As a key issue we address the full-glacial presence of trees in Europe north of the Mediterranean region, a widely debated issue with evidence for and against emerging from several research fields and data sources. We compiled and analyzed a database of bird fossil occurrences from archaeological sites throughout the Western Palearctic and spanning the Saalian-Eemian-Weichselian stages, i.e. 190,000–10,000 years BP. In general, cold and dry-adapted species dominated these late Middle Pleistocene and Late Pleistocene fossil assemblages, with clear shifts of northern species southwards during glacials, as well as northwards and westwards shifts of open-vegetation species from the south and east, respectively and downwards shifts of alpine species. A direct link to climate was clear in Northwestern Europe. However, in general, bird assemblages more strongly reflected vegetation changes, underscoring their usefulness for inferring the vegetation structure of past landscapes. Forest-adapted birds were found in continuous high proportions throughout the study period, providing support for the presence of trees north of the Alps, even during full-glacial stages. Furthermore, the results suggest forest-dominated but partially open Eemian landscapes in the Western Palearctic, including the Northwestern European subregion.

## Introduction

A main response of species to climatic changes has been to move by niche tracking i.e. following the shifting climate to remain in favorable living conditions. Numerous examples of this response are reported in the literature; both from the present and past, on short time scales and on long geological time scales, and covering a wide range of organism groups [1]–[9]. Studies of past communities and range shifts provide important insights for understanding and predicting current and future biotic responses to the ongoing global warming and for guiding conservation management in the face of climate change (cf. [10]).

Past climatic cycles of the Quaternary have had great impact on species ranges. The Late Pleistocene in the Western Palearctic has attracted much attention in relation to the effects of climate change on biotic dynamics. This period covers the Last (Eemian) Interglacial and the Last (Weichselian) Glacial terminating with the beginning of the present interglacial (Holocene). Stable oxygen isotope variations in ice cores and deep-sea sediments have provided a detailed record of past changes in global climate [11]–[13], but regional vegetation responses to these changes remains a matter of debate. Generally the vegetation oscillated on a north-south and east-west gradient between two vegetation extremes; coniferous and deciduous forests under warm, oceanic conditions, and open subarctic steppe-tundra under cold, continental conditions [14]. Paleontological studies indicate that many organisms shifted southwards during glacial periods, as a response to cold temperatures and aridification [15]. Similarly, studies on present range shifts show that the opposite is happening now in response to current global warming [4], [16]. Palynological evidence show that during the warm Eemian there was a drop in open vegetation and an increase of forest, which terminated with an increasing dominance of first cold-tolerant tree species and finally herbaceous species, marking the breakup of European forests in response to the beginning of colder conditions [17]–[20]. This view of a forest-covered interglacial Europe is widely accepted, albeit the exact vegetation structure and the degree of openness is debated (e.g. [21]). The extent of forest during the cold stages of the Weichselian has attracted more attention from paleoecologists. The focal point of discussion is the contradictory evidence from pollen studies versus macrofossil studies and climate reconstruction models. Traditionally, most forest species were believed to have survived the full-glacial periods in mountainous belts of favorable conditions in the Iberian, Italian and Balkan peninsulas, the so-called glacial refugia hypothesis [22]–[25]. This was supported by palynological studies indicating an absence of forest north of the Alps [18], [26], even during some of the milder periods [20], and phylogeographic studies reporting genetic patterns indicating isolation of temperate species in the southern refugia followed by post-glacial recolonization of the north [27], [28]. This traditional view has been challenged in recent years by tree charcoal remains in Central and Eastern Europe, indicating the presence of boreal forest tree species and even thermophilous deciduous tree species during full-glacial conditions [29], [30]. The presence of more northerly glacial forests is supported by other studies on plant [31] and vertebrate fossils [32], as well as genetic evidence from boreal trees [33] and temperate animals [34], as well as distribution hind-casting and vegetation simulation studies, suggesting a possibility for the presence of tree species at mid-to-high latitudes in Europe even during the Last Glacial Maximum (LGM) [35], [36] . Notably, forest tree species could have survived at low densities in discontinuous so-called cryptic refugia [25], [31], [37].

This study adds to these current discussions on the European Late Pleistocene by investigating the paleoecological implications of the Western Palearctic avian fossil record, with birds being a little-studied group in this context. Some earlier studies using avian fossils as proxies for past environments exist, but mainly on local scales [38]–[43], albeit with some regional-scale studies [44]–[47]. By assuming that stratigraphic bird assemblages reflect the relative weight of different biotypes over time, most of these studies have provided snapshots of local environments in a given time and a continental-scale paleoecological study remains to be done. The use of birds as proxies has several advantages: 1) They are mostly easily identified to species level due to morphological skeletal characteristics [48], [49], though passerines are often perceived as a group where species-level identification can be difficult. 2) Most birds respond mainly to auditory and visual stimulants in a relatively similar manner as humans, making habitat parameters easily defined [48], an advantage that is strengthened by the fact that most bird species have specific – and in the Western Palearctic, well-known – habitat and often vegetation-defined requirements [46]. 3) The great mobility of most bird species furthermore make them more likely to fill their potential range relatively well as they can more easily track habitat shifts caused by climate change, than many less mobile organism groups with slower migration rates [50]. The main focus in this study is on the extent to which climate-driven range shifts have occurred in the period 190,000–10,000 years BP, either directly via abiotic effects or indirectly via vegetation effects. This period covers the Eemian and Weichselian as well as the latest part of the penultimate glacial, the Saalian (190,000–130,000 years BP) as well as the earliest Holocene (11,700–10,000 years BP). Furthermore, the implications for the much-discussed issues of the extent of forest cover in Europe during full-glacial periods and – vice versa – the degree of vegetation openness during the interglacial are also considered. The following specific questions are investigated: Firstly, is there direct evidence for range shifts in the Western Palearctic avifauna in the past Saalian-Eemian-Weichselian time span, as seen in other organism groups? Secondly, to what extent can these shifts be linked to the known past changes in climate and vegetation? More specifically, species associated with cold, dry and open vegetation environments are expected to dominate assemblages from glacial stages, while species of warm, humid and wooded environments are expected to dominate during the interglacial stage. Thirdly, how does the evidence provided by the avian fossil record relate to the ongoing debate on the degree of woodland presence outside traditional southern glacial refugia during the glacial stages? Fourth and lastly, bird compositional dynamics and questions 1–3are also investigated for the Northwestern European subregion within the Western Palearctic. There are clear subregional differences in the strength of the glacial-interglacial climate changes, and the Northwestern subregion has experienced relatively large climatic fluctuations during the Late Pleistocene [24], [36], [51]. How has these changes affected the bird communities in this area compared to the entire Western Palearctic?

## Methods

### Database

A database on Western Palearctic bird fossils for the last 190,000–10,000 years BP was compiled based on the information contained in the monograph “Pleistocene Birds of the Palearctic: A Catalogue” and its revised supplement by Tyrberg [52], [53]. This collection makes up the most extensive synthesis of avian fossils in Pleistocene Europe and contains information on fossiliferous sites collected from a large quantity of original work and secondary references.

**Figure 1:**
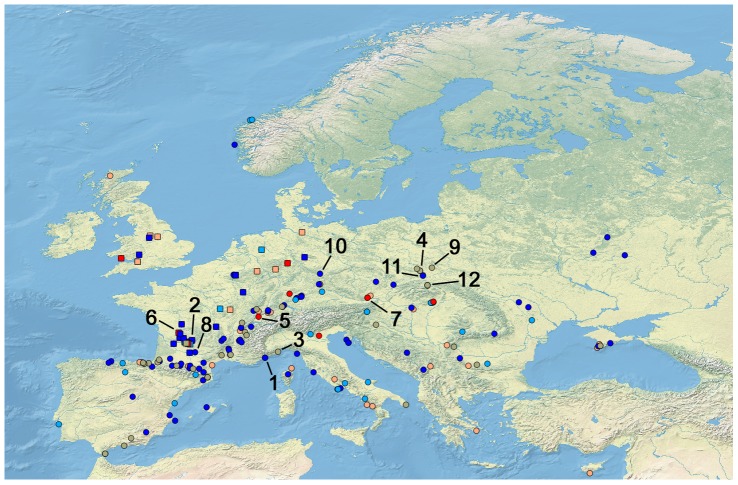
Position of sites included in the database. All sites are included in the statistical analyses of the bioclimatic properties of birds of the Western Palearctic, while only square sites are included in the analyses of the Northwestern subregion. The color of each site corresponds to the temperature trend of the stage it has been dated to, inferred from Table 1; Dark red = MIS 5, Light red = MIS 1/2, Light blue = MIS 3–4, Dark blue = MIS 2 and 6, Grey = Sites with multiple stratigraphic layers assigned to different stages. Numbers designate sites specifically mentioned in the text: 1) Grotte du Lazaret, France, 2) La Fage, France, 3) Arene Candide, Italy, 4) Krucza Skala, Poland, 5) Grotte de Cotencher, Switzerland, 6) Fontéchevade, France, 7) Schusterlucke Cave, Austria, 8) Abri de Fontales/Saint-Antonin Noble-Val, France, 9) Raj Cave, Poland, 10) Zwergloch, Germany, 11) Mamutowa, Poland, 12) Oblazowa Cave, Poland.

The database was comprised of information on the Western Palearctic, defined as Europe south to the Mediterranean and east along the Caucasus, bordered to the west by the Atlantic Ocean and to the east by the 40 degree longitude. Coordinates for each site was obtained through extensive internet and journal searches, and compared with the maps provided by Tyrberg [52]. They were recorded as decimal degrees and mapped using Quantum GIS version 1.8.0 with a European base map obtained from Natural Earth [54] (Fig. 1). The sites and sometimes even individual stratigraphic layers of sites included in Tyrberg's catalogues have been dated using a variety of different methods ranging from absolute radiometric dating (such as 14-C and U/Th) to relative dating using stratigraphic and archaeological methods. Consequently, care was taken to infer calendar dates for the sites used in the database and 14-C calendar dates were calibrated using CalPal-2007^online^ [55]. For the purpose of subsequent analyses, the sites were then categorized according to the Marine Isotope Stages (MIS's) defined in Table 1. These definitions were used to hinder the appearance of individual site entries in more than one category in the database, as some sites would otherwise have dating estimates that overlapped more than one MIS category. The Late-Glacial border category between MIS 1 and 2 (denoted “1/2“) was created in order to further accommodate this transition period, notably because many sites clustered within this short period which contain much warmer episodes than the rest of MIS 2. Furthermore, due to small sample sizes, the moderately cold MIS 3 and 4 as well as the relatively warm MIS 5a to 5e (the Last Interglacial) were pooled together as MIS 3-4 and MIS 5, respectively. Individual stratigraphic layers in the catalogues, with dating estimates that overlapped multiple stages of opposite climatic regimes (e.g. spanning MIS 5a and MIS 4) were excluded. We also excluded layers older than 190,000 and younger than 10,000 years BP due to scarcity of the former and the sporadic coverage of the latter in the source material.

**Table 1:** Marine Isotope Stage definitions and sample sizes in the study.

MIS	From (yr BP)	To (yr BP)	Sample size Western Palearctic	Sample size northwestern Europe	Temperature characteristics
1/2	13,000	10,000	95	8	Transition, cold to warm
2	28,000	10,000	181	17	Fully glacial
3–4	75,000	28,000	97	9	Cool
5	130,000	75,000	12	5	Warm, Fully interglacial
6	190,000	130,000	10	-	Fully glacial

MIS definitions was made loosely under influence by Taylor & Aitken [89], though constricted by sample size issues. The short transitional period MIS 1/2 was created due to a high concentration of sites dated to this time and is overlapped by a few sites in MIS 2 that have large date range estimates, causing both MIS categories to have the same end date. Sample size states the number of individual sites or stratigraphic layers for each period included in the analyses of the Western Palearctic and Northwestern European subregion, respectively.

The fossil bird species for each site were indexed in the database as follows: Fossils that had been taxonomically identified to a single species were scored a 2 and fossils that had not been confidently identified (e.g. denoted “?”, “cf.” or “aff.”) were scored a 1. Fossils identified as belonging to one of two or more closely related species were scored with a 1 for each species (e.g. *Corvus corone*/*frugileus*). Fossils that had only been identified to family level or higher were excluded and fossils identified to subspecies were scored under their respective species. 16 non-extant species were excluded given uncertainty regarding their climatic and habitat requirements. As the exception, the recently exterminated *Pinguinus impennis* (Great Auk) was not excluded as its requirements are relatively well-known. Furthermore, only sites and stratigraphic layers containing a minimum of 10 species were included in the database. This was done in order to minimize errors by focusing on sites where birds had received clear attention and to reduce stochastic noise due to small sample size. The individual species were categorized according to their present distributional association with three climate and habitat attributes, namely temperature, humidity and vegetation, as defined by Finlayson [14]; Temperature: According to Finlayson each species' breeding distribution was compared to a bioclimatic map of the World and given a rank on a temperature gradient based on which bioclimatic area it occupies. The gradient went from 1% indicating the coldest conditions to 100% indicating the warmest conditions. From this rank, each species was classified in a group from A to E: A = 1–20%, B = 21–40%, C = 41–60%, D = 61–80%, E = 81–100%. Humidity: As with temperature, this gradient went from 1% indicating the most xeric conditions to 100% indicating the most humid conditions: A = 1–20%, B = 21–40%, C = 41–60%, D = 61–80% and E = 81–100%. Vegetation: We focused on foraging habitat rather than nesting habitat, since the source material, did not indicate maturity of the fossil individuals and therefore there was no way of knowing if a fossil came from a breeding site or not. The classifications were: F = Forest, with a high density of trees; O = Open; M = Mixed, including savannah, scrubland and tree-open-habitat-mosaics; R = Rocky; W = Wetland, all kinds except marine; Ma = Marine; A = Aerial. Of these habitat categories provided by Finlayson, only forest, mixed and open were used here to infer temporal changes in vegetation. Twelve species in the database were not included in Finlayson's work and habitat information for these were obtained from the Birdlife International webpage [56] and classified according to the habitat categories provided by Finlayson [14]. These species were excluded from the climate-related part of the analyses.

The final database consisted of 361 species from 61 families distributed among 227 fossil sites on a total of 474 stratigraphic layers (Dataset S1 in Supporting Information).

### Analysis

To investigate if the fossil record show evidence of species range shifts during the 190,000–10,000 years BP period or not, we compared the distribution of fossil occurrences to species' present-day distribution as estimated by Birdlife International and NatureServe [57]. Many species have distributions covering large latitudinal spans suggesting high bioclimatic tolerances [14]. Given that fossils represent presence-only data so that observed absences cannot with certainty be inferred as true absences, range shifts for such widely distributed species cannot easily be concluded with certainty. We therefore focused on species with present ranges restricted to more localized areas when assessing range shifts.

To explore climate and vegetation associations of the fossil bird species in relation to different stages, the species index number for each site in the database was summarized and from this, the proportions of birds within each temperature, humidity and vegetation category were calculated. A few sites consisted of more than 10 stratigraphic layers dated to the same MIS and proportions for these were averaged in order to avoid pseudoreplication. Grotte du Lazaret (FR147, FR148), La Fage (FR171), sites dated to MIS 2 of Arene Candide (IT3) and Krucza Skala (PO37) with 20, 11, 17, 16 and 17 layers respectively, were all averaged (Fig. 1: site 1–4). Using R Studio (version 0.97.332) Kruskal-Wallis one-way analyses of variances between means of proportions of the different MIS's were performed for each climate and vegetation category followed by posthoc Wilcoxon rank sum tests.

The above questions were re-assessed just for Northwestern Europe by repeating the statistical analyses described above using entries in the database from sites of Belgium, non-alpine Germany, non-alpine and non-Pyrenean France, the Netherlands and England only (Fig. 1). MIS 6 were excluded in this part of the analysis due to low sample size.

To assess the robustness of the results to uncertainties in the identification of fossils to species level, two supplementary analyses were made. One in which each species' habitat and climate attribute were replaced by the average of the genus (AvGenus) and one in which Passeriformes were excluded from the analysis (NoPas), as this group is probably the most challenging for species-level identifications. All of the analyses described above were repeated on these alternative datasets.

## Results

### Range Shifts

**Figure 2:**
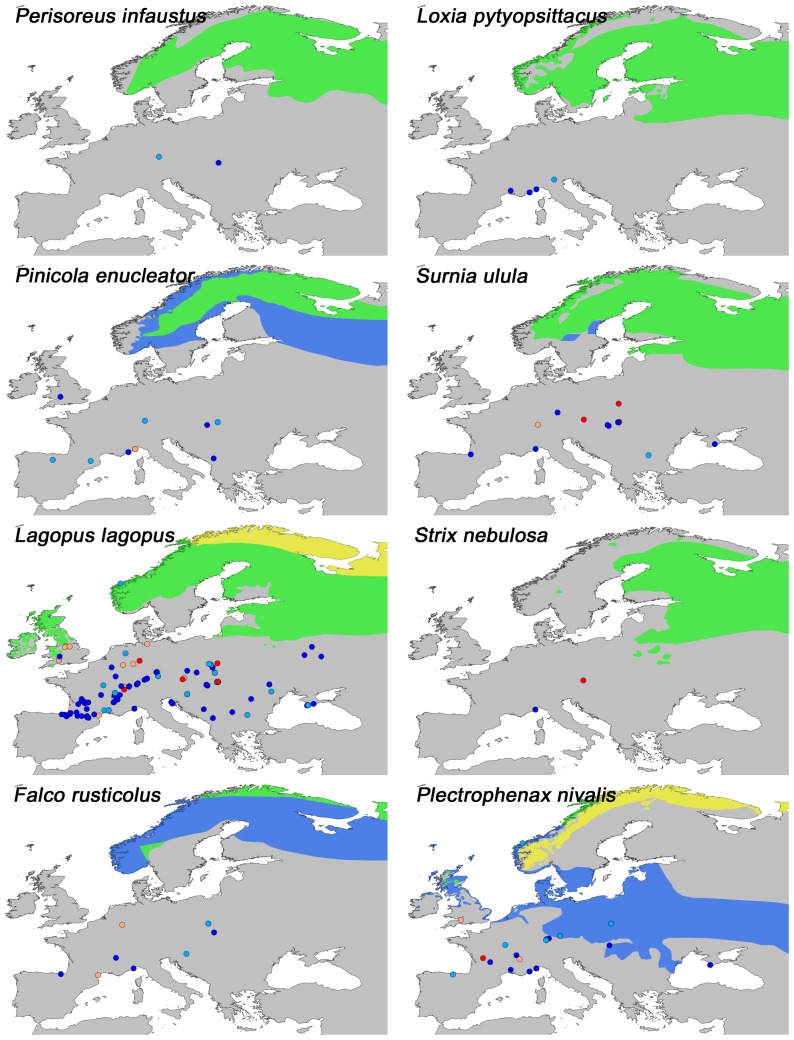
Present distribution and fossil locations of selected species in the analysis I. Present distribution is indicated by native resident (Green), native breeding (Yellow) and native non-breeding (Blue) ranges. For explanation on color codes for fossils sites see Fig. 1. These species all have fossil occurrences further south of their present distribution indicating latitudinal range change in the past.

**Figure 3:**
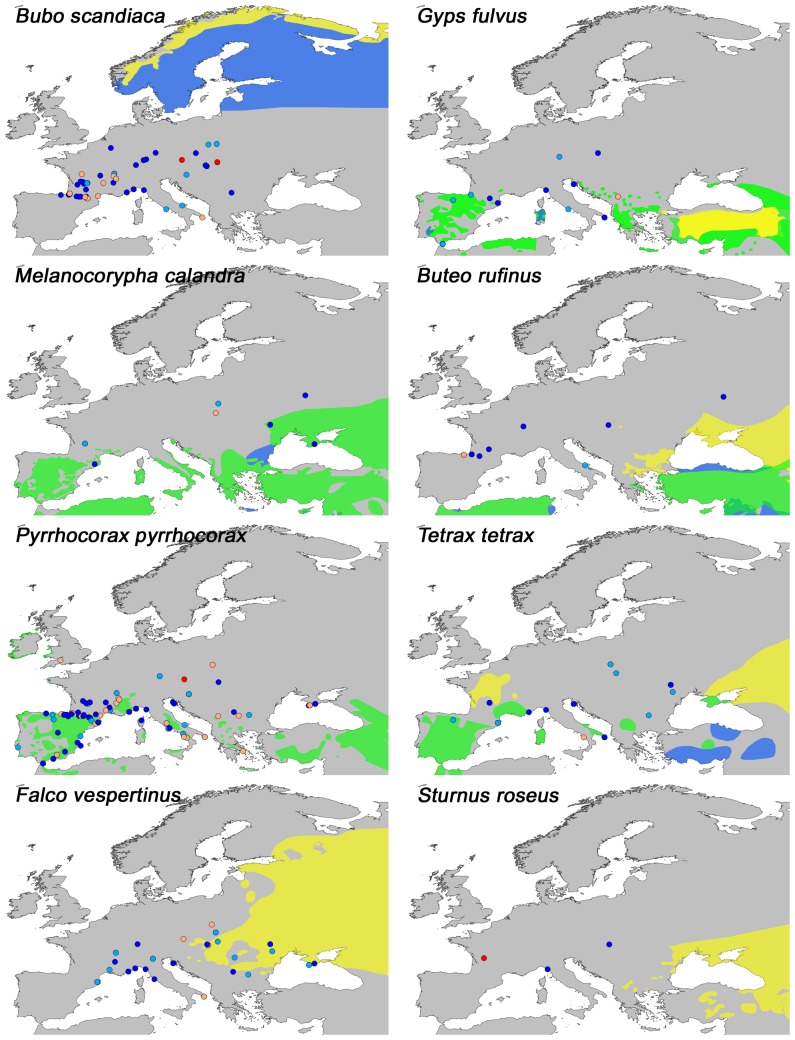
Present distribution and fossil locations of selected species in the analysis II. For explanation on distribution area, see Fig. 2. For explanation on color codes for fossils sites see Fig. 1. These species have fossil occurrences north (south in the case of *Bubo scandiaca*) of their present distribution indicating latitudinal range change in the past, except for *Sturnus roseus* that exhibits longitudinal range change.

**Figure 4:**
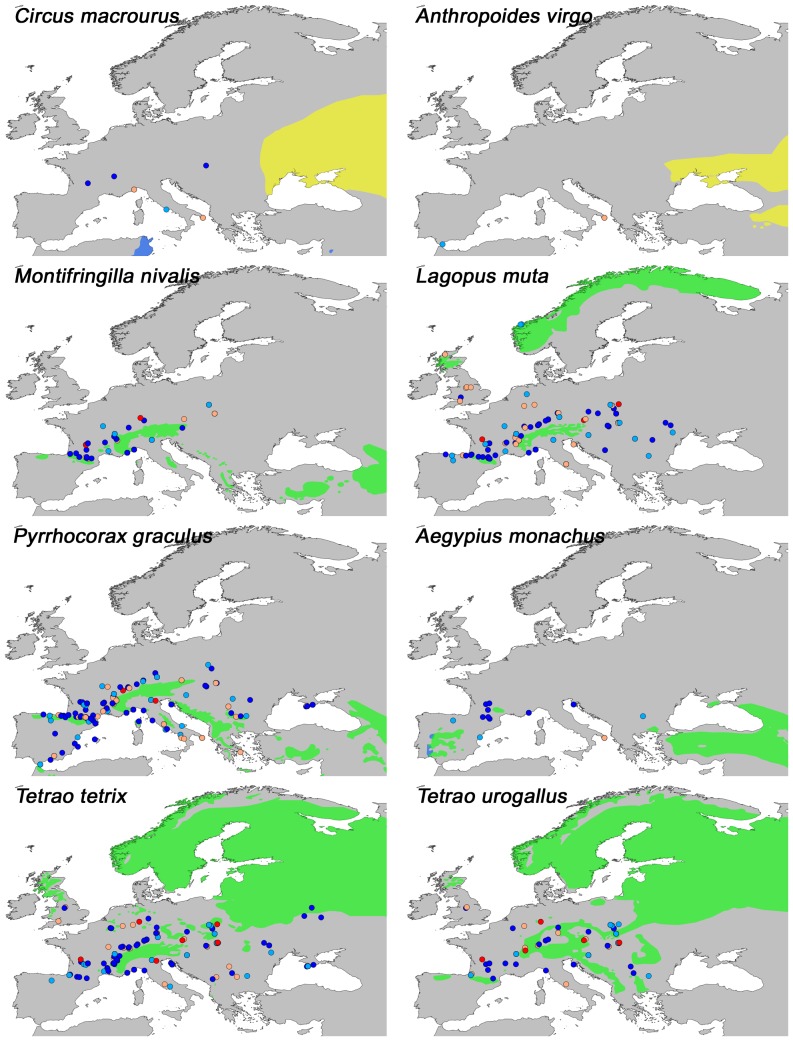
Present distribution and fossil locations of selected species in the analysis III. For explanation on distribution area, see Fig. 2. For explanation on color codes for fossils sites see Fig. 1. These species exhibit westwards longitudinal (*Circus macrourus and Anthropoides virgo*) and downwards altitudinal (*Montifringilla nivalis, Lagopus muta and Pyrrhocorax graculus*) range changes as well as evidence of past larger ranges relative to today (*Aegypious monachus, Tetrao tetrix and Tetrao urogallus*).

A number of latitudinal shifts in range are apparent. Southward glacial shifts are found for the forest species *Perisoreus infaustus* (Siberian Jay), *Loxia pytyopsittacus* (Parrot Crossbill), *Pinicola enucleator* (Pine Grosbeak), *Surnia ulula* (Northern Hawk Owl) and *Strix nebulosa* (Great Grey Owl), as well as for the open vegetation species *Lagopus lagopus* (Willow Ptarmigan), *Falco rusticolus* (Gyr Falcon), *Plectrophenax nivalis* (Snow Bunting) (Fig. 2) and *Bubo scandiaca* (Snowy Owl) (Fig. 3). All fossils of these species are from cold stages or stadials, except from one occurrence of *Lagopus lagopus* at Grotte de Cotencher (CH10) (Fig. 1: site 5) dated to the Odderade interstadial. Northward shifts under both cold and warm stages, are seen in *Gyps fulvus* (Griffon Vulture), *Melanocorypha calandra* (Calandra Lark), *Buteo rufinus* (Long-legged Buzzard), *Pyrrhocorax pyrrhocorax* (Red-billed Chough) and *Tetrax tetrax* (Little Bustard) (Fig. 3). Some species also exhibited longitudinal range shifts, with a tendency for westward expansions relative to today, e.g. *Falco vespertinus* (Red-footed Falcon), *Sturnus roseus* (Rosy Starling) (Fig. 3), *Circus macrourus* (Pallid Harrier) and *Anthropoides virgo* (Demoiselle Crane) (Fig. 4), which are all open-vegetation species. Fossils of these species have been dated to belong to cold stages, the Late-Glacial and to the Eemian. Furthermore, downwards altitudinal range shifts relative to today are seen for *Pyrrhocorax graculus* (Yellow-billed Chough), *Lagopus muta* (Rock Ptarmigan) and *Montrifringilla nivalis* (White-winged Snowfinch) (Fig. 4). Most of these fossils are from cold stages or stadials, except for a single occurrence of *Lagopus muta* at the Eemian-dated site Fontéchevade cave (FR84) (Fig. 1 :site 6). Lastly, *Aegypius monachus* (Cinereous Vulture), *Tetrao urogallus* (Western Capercaillie) and *Tetrao tetrix* (Black Grouse) that today have patchy or disjunct distributions have fossil occurrences indicative of widespread occurrences in the past (Fig. 4).

### Climate And Vegetation-Related Patterns For The Western Palearctic

**Figure 5:**
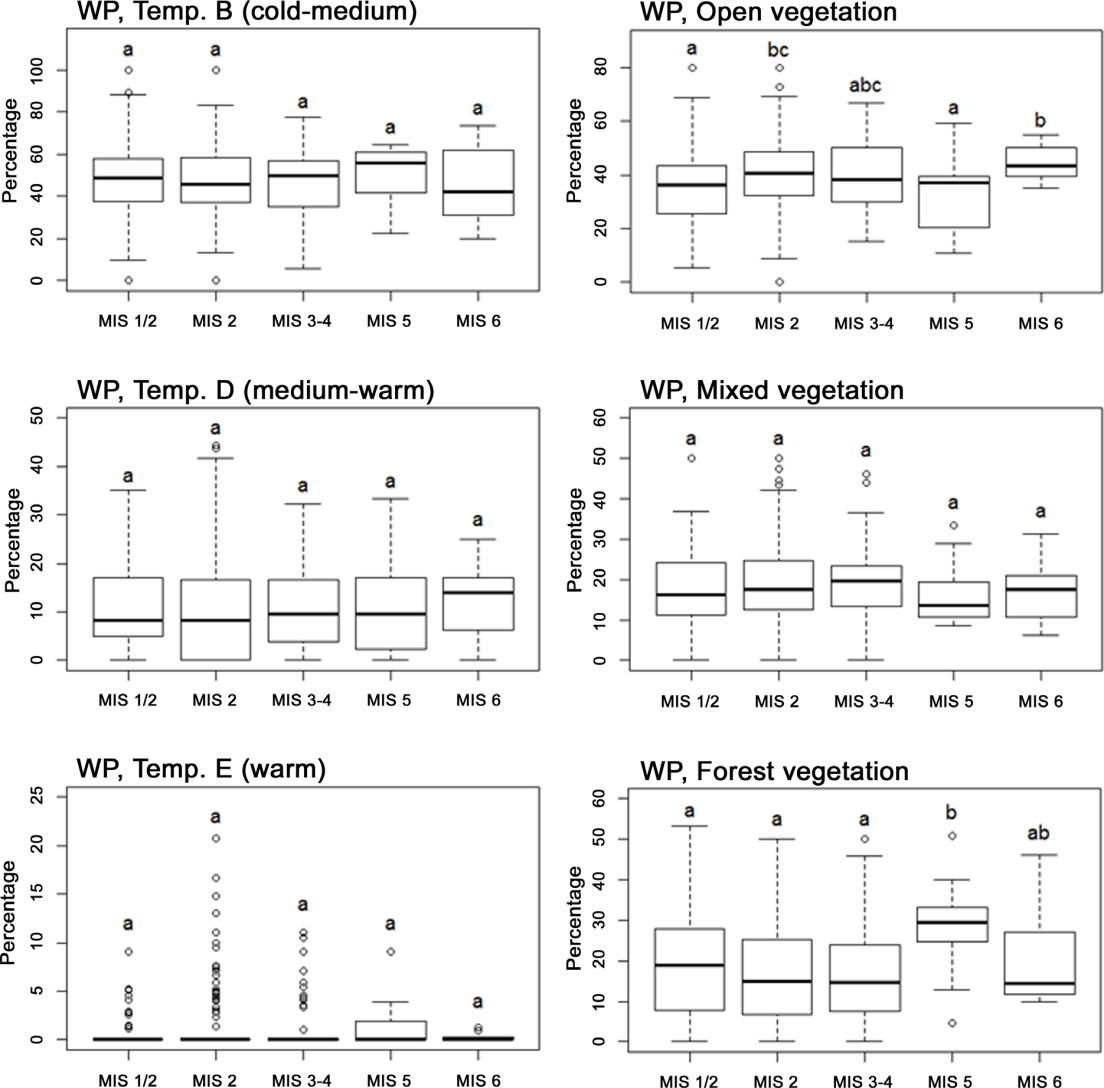
Boxplots of vegetation and temperature properties for birds of the Western Palearctic (WP). Boxplots shows the percentage of fossil bird species preferring cold-medium (Temp. B), medium-warm (Temp. D) and warm (Temp. E) conditions (Left) and open, mixed and forest vegetation (Right) in the Western Palearctic, for each MIS. Boxes show the median, 25th and 75th percentile and whiskers extending 1.5 interquartile range (IQR). Dot symbols identify outliers. Letters indicate significant relationships according to Wilcoxon signed-rank tests (p≤0.05). Open and Forest vegetation are the only significant variables according to variance analyses (Table 2) and show significant higher proportions of open vegetation species during the glacial periods MIS 2 and 6 compared with the partly interglacial MIS 5 and the transitional period MIS 1/2. Furthermore, MIS 5 had significantly higher proportions of forest species compared with later periods of colder temperatures.

Avian communities were dominated by cold-adapted species throughout the 190,000–10,000 years BP period (Table S1 in Supporting Information), with species of temperature categories A and B constituting more than half of the fossils. Similarly, there was also a general dominance of xeric-adapted species consistent with the cold, dry glacial climate. Species of open vegetation fluctuated in proportion between cold (average of MIS 2, 3–4 and 6) and warm (MIS 5) stages with 41.8% and 32.6%, respectively. For forest species the proportions were 18.0% for average cold stages and 28.5% for the warm stage (Table S1). Statistically, the strongest differences between stages were between open and forest vegetation categories, while there were no significant differences among climate categories (Table 2 and Fig. 5). Open-vegetation species constituted higher proportions of assemblages during the two full-glacial stages, MIS 2 and 6, compared with the warm and partly interglacial MIS 5 and the Late-Glacial MIS 1/2, while forest species conversely peaked during MIS 5. The temporal distributions of proportions were similar in the two supplementary analyses (Table S2, Fig. S1, Table S3 and Fig. S3), indicating that these findings should not be sensitive to species-level identification uncertainties.

**Table 2:** Results of Kruskal–Wallis one-way analysis of variance.

	Western Palearctic			Northwestern Europe		
Variable	*χ* ^2^	Df	p	*χ* ^2^	Df	p
Temperature A	3.1243	4	0.5372	0.8493	3	0.9317
Temperature B	1.5118	4	0.8246	11.3243	3	**0.02315**
Temperature C	4.2025	4	0.3793	4.659	3	0.3241
Temperature D	2.8698	4	0.5799	14.4951	3	**0.005872**
Temperature E	3.0851	4	0.5437	16.437	3	**0.002486**
Humidity A	8.6433	4	0.0707	3.1268	3	0.5368
Humidity B	5.2386	4	0.2637	1.8566	3	0.7621
Humidity C	3.0629	4	0.5474	3.5202	3	0.4748
Humidity D	5.6540	4	0.2265	1.7719	3	0.7776
Humidity E	6.7712	4	0.1485	2.1658	3	0.7053
Vegetation Open	14.159	4	**0.0068**	8.2334	3	0.08339
Vegetation Mixed	2.9086	4	0.5732	4.3537	3	0.3602
Vegetation Forest	10.7363	4	**0.0297**	11.015	3	**0.0264**

χ^2^ = value of H statistic for the adopted significance level; Df = degree of freedom; p = significance level (p≤0.05). Bold values indicate significance. The table is divided between the Western Palearctic and the Northwestern European subregion and shows the results of variance analyses between means of MIS's for several temperature, humidity and vegetation categories and is based on the proportions of fossil bird species assigned to each category. Temperature categories are based on a gradient going from the coldest (Temperature A) to the warmest (Temperature E) conditions and humidity follows a similar gradient going from the driest (Humidity A) to the wettest (Humidity E) conditions.

### Climate And Vegetation-Related Patterns For The Northwestern Subregion

**Figure 6:**
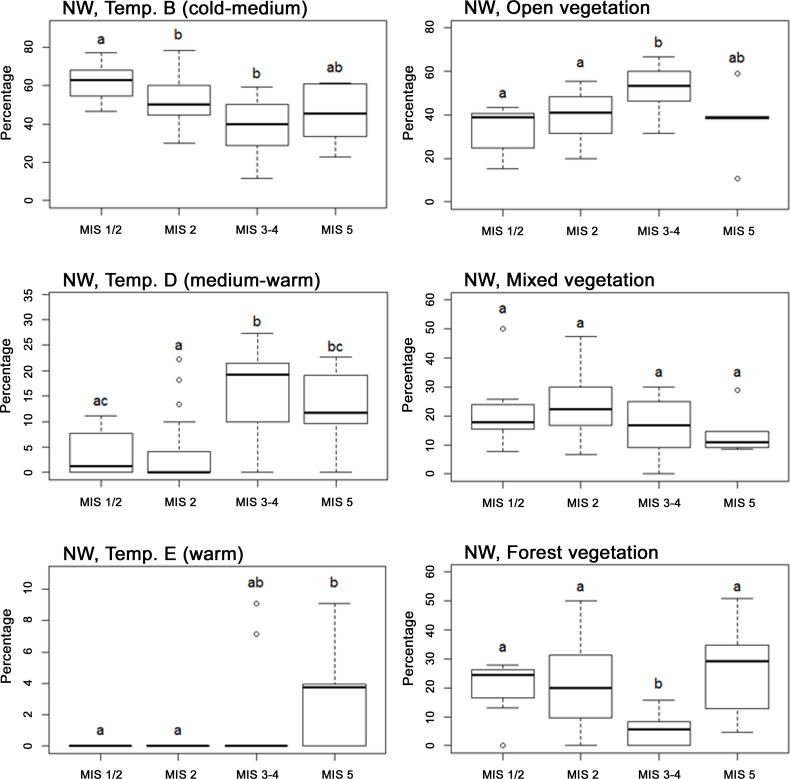
Boxplots of vegetation and temperature properties for birds of the Northwestern European subregion (NW). Boxplots shows the percentage of fossil bird species preferring cold-medium (Temp. B), medium-warm (Temp. D) and warm (Temp. E) conditions (Left) and open, mixed and forest vegetation (Right), in Northwestern Europe, for each MIS. Boxes show the median, 25th and 75th percentile and whiskers extending 1.5 interquartile range (IQR). Dot symbols identify outliers. Letters indicate significant relationships according to Wilcoxon signed-rank tests (p≤ 0.05). In this analysis the three temperature variables presented here, as well as the forest vegetation variable were significant according to variance analyses (Table 2). Species of warmer temperatures are present in larger proportions at MIS 5 and to some degree MIS 3-4, while cold adapted species are more prominent in MIS 1/2. The vegetation trends follow that of the Western Palearctic.

In contrast to the patterns for the Western Palearctic, considering only Northwestern Europe, bird communities exhibited significant differences between stages in terms of temperature associations (Table 2, Fig. 6). Overall, warm-adapted species predominated during MIS 5 and 3–4, while cold-adapted species predominated during MIS 2 and 1/2. Of the vegetation categories, the only significant difference was that MIS 3–4 had a smaller proportion of forest-associated bird species than other periods. The results of the supplementary analyses were overall consistent with these results (Table S2, Fig. S2, Table S3 and Fig. S4).

## Discussion

Numerous bird species have experienced range shifts in the Western Palearctic over the 180,000 years study period from the late Middle Pleistocene to the Late Pleistocene/Holocene transition. These shifts can primarily be linked to vegetation changes in response to climate change, with a dominance of species of open vegetation during cold periods and forest-adapted species during warm periods. However, there was a strong link to climate per se in the more climatically exposed subregion; Northwestern Europe.

### Avian Responses To Climate Change

Studies on avifaunal responses to recent climate change have provided insight into the effects of change on bird species distribution and community composition. Generally, the strongest effects are found with regard to phenology and population dynamics, though shifts in range are also apparent [58]. A local study in California, reported range changes for several bird species over the course of a century in response to temperature and precipitation changes [59], and a study in England found climate change effects even within a short time span of 20 years [60]. Given the emerging present trends, it is not surprising to find that range shifts have occurred in response to the dramatic Quaternary glacial-interglacial changes in climate and vegetation, as reported here. Such shifts were especially evident for species with a presently northern distribution (Fig. 2). During periods of the Last Glacial, when Northern Europe was inhospitable due to severe climate and extensive glaciation, these species were forced south to Central and Southern Europe. The opposite scenario in which birds of the south move northwards during warm stages is not nearly as clear, at least with respect to temperature responses per se (Fig. 3). Instead many of the observed northwards extensions are from cold periods as well. Apart from *Pyrrhocorax pyrrhocorax*, these latter species are all dry-adapted and associated with open-vegetation, matching the general environment that existed during the cold stages and it is probably aridity and openness rather than temperature that allowed these species to spread northwards. Similar glacial expansions of tundra and steppe species have also been observed for plants, insects and mammals [61]–[65]. The rich megafauna of the glacial steppe-tundra would have provided ample food for the scavengers such as *Gyps fulvus* (Fig. 3) and *Aegypius monachus* (Fig. 4), providing an explanation for the larger ranges observed for these species in the past, similar to what have been proposed for the large range changes observed for Late Pleistocene North American scavenging birds [66]–[68]. Evidence of longitudinal range shifts as a response to glaciations has previously been shown for beetles [7], [62] and mammals [69] and is generally seen along a longitudinal moisture gradient, which in Europe is created by a strong west-east precipitation gradient. It is thus likely that the longitudinal range shifts observed here for birds are also due to the increased aridity and openness discussed above. This climate would have also favored alpine species and the downward expansion of *Montifringilla nivalis, Lagopus muta*, *Pyrrhocorax graculus* and *Pyrrhocorax pyrrhocorax* into the European lowlands is consistent with this (Fig. 3 and Fig. 4). The present disjunct distributions of these species thereby exemplify southern topographic refugia for cold-adapted species during interglacials, a phenomenon also observed in plants and mammals [22], [25], [70]. Finally we note that a lot of species did not exhibit marked past range shifts, indicating that they were not strongly affected by the climate changes and were able to survive in place.

The Late Pleistocene avifaunal range shifts nicely illustrate individualistic responses to environmental changes, as broadly reported for other organism groups [9], [28], [71]. These species-specific responses probably reflect that the individual species are limited by different abiotic factors and biotic interactions as well as having varying dispersal abilities. An important outcome of this is the creation of non-analogue assemblages, which is also seen in the Late Pleistocene avifaunal record. In Schusterlucke cave (AU16) (Fig. 1: site 7), dated ca. 115,000 years BP, for example, *Surnia ulula* and other northern species like *Lagopus lagopus*, *Bubo scandiaca* and *Strix nebulosa* are found together with South European mountain species like *Prunella collaris* (Alpine Accentor), *Pyrrhocorax graculus* and *Pyrrhocorax pyrrhocorax* and temperate species like *Perdix perdix* (Grey Partridge), *Corturnix coturnix* (Common Quail), *Crex crex* (Corncrake), *Cuculus canorus* (Common Cuckoo) and *Picus viridis* (Eurasian Green Woodpecker). Similarly, 100,000 years later during the Late-Glacial in France at Abri de Fontalés cave (FR16) (Fig. 1: site 8) *Lagopus lagopus* and *Bubo scandiaca* are also found together with *Pyrrhocorax graculus*, *Prunella collaris*, *Perdix perdix* and *Hirundo rupestris* (Eurasian Crag-martin). The examples from Tyrberg's catalogue are numerous and indicate that such non-analogue assemblages were not rare occurrences. This phenomenon has also been observed for other taxa [72], [73] and has important implications for both paleoecology and future ecological forecasting [74].

An important caveat to this interpretation is that some localities may sample from time periods spanning varying climates or contain mixed stratigraphic layers. For example, the apparent occurrences of cold-adapted species like *Lagopus muta*, *Buteo lagopus* and *Plectrophenax nivalis* at the Eemian site Fontéchevade Cave (FR84) (Fig. 1: site 6) together with species like *Sturnus roseus*, *Hirundo rupestris* and *Coturnix coturnix* and others associated with a high to medium temperature could reflect mixing of the stratigraphic layers [75].

Bird species preferences for temperature and humidity did not differ significantly between stages, when considering the entire Western Palearctic (Table 2), perhaps reflecting that this broad region spans much climatic heterogeneity and encompasses both climatically unstable and relatively stable areas, buffering against overall changes. In Northwestern Europe, however, the proportion of birds in the different temperature preference categories differed between stages (Table 2 and Fig. 6), indicating that this smaller subregion did not have the sufficient conditions for all climatic types to persist through all stages. A similar heterogeneous pattern in community composition between stages has been found for Middle Pleistocene mammals, with marked shifts in Northern Europe due to the strongly disruptive effects of glacial climate here and continuity in Southern Europe [76]. In contrast to the patterns for climate preferences, there were clear shifts in general vegetation preferences between stages both for the Western Palearctic overall and for Northwestern Europe (Table 2, Fig. 5 and Fig. 6). In Northwestern Europe, the proportion of forest species during MIS 3-4 is surprisingly small compared to the colder, more arid, MIS 2 (Fig. 6). This pattern is probably artefactual, reflecting that most of the MIS 2 data belong to the end of the period when there were several warm episodes. Another interesting pattern that emerged is the high proportion of warm-adapted (Temp. D) birds in combination with the low proportion of forest-adapted birds during MIS 3–4 in Northwestern Europe (Fig. 6). This suggests that although temperatures were relatively high (a majority of sites within this category dates to MIS 3, the warmer of the two stages), tree species were not able to colonize Northwestern Europe at this time, possibly due to migration lag, as has been suggested by Coope based on fossil beetles [77]. Lags in treeline shifts and tree species ranges in response to climate change are widely reported in the literature [10]. The continuous presence of open, mixed and forest-adapted species throughout the study period are in agreement with simulation studies that found a similar continuous presence of both non-arboreal and different arboreal vegetation types, throughout the middle to late Weichselian in Europe [35], [36] and the direct fossil evidence for the presence of both boreal and temperate tree species in Central and Eastern Europe [30].

### Vegetation Implications Of The Avian Fossil Record

The current debate on the degree of forest cover during the full-glacial and interglacial stages is treated in detail in the following by considering the observed stage-by-stage pattern in forest- and open-habitat-associated bird species, as well as the more specific habitat requirements of the various species found.

**Figure 7:**
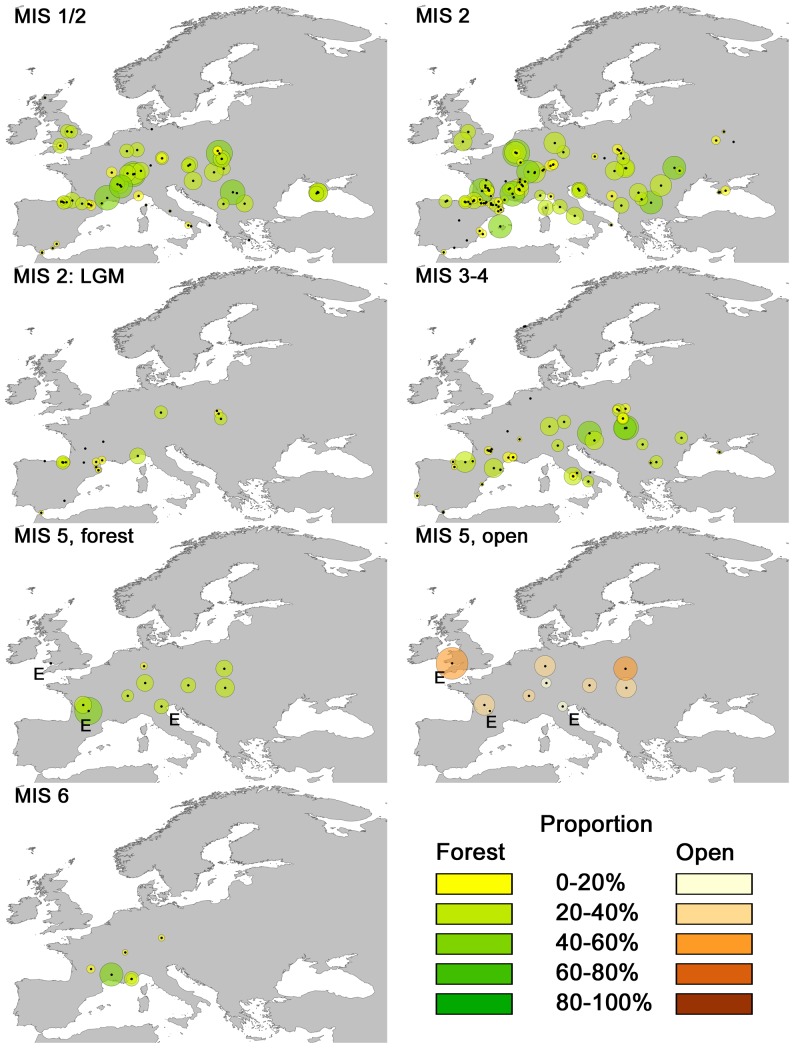
Spatial distribution of site proportions of forest bird species for each MIS in the study. The size and color scales of individual sites indicate the proportion of forest species for each site. Centers are marked with a black dot. E denotes sites of Eemian origin. The maps show that the sites with the highest proportion of forest birds are found within the warm MIS 5, but that MIS 1/2, 2 and 3–4 all have considerable proportions as well despite their overall cold temperature trends. Fossils from the LGM (22,000–18,000 years BP) within MIS 2 show that forest species were present in Poland and Germany as well as the Mediterranean. In addition, the proportion of open vegetation species is shown for MIS 5 indicating that there was a high degree of openness in the landscape, also during the Eemian.

The Saalian glacial stage (MIS 6) surpassed the Weichselian both in ice sheet extension and duration [78]. Permafrost prevailed north of the Alps and the vegetation here was mainly a steppe-tundra mixture, probably reflecting the low temperatures and low CO_2_ levels [78]. Palynological evidence suggest that tree species were mainly found south of the Alps in localized refugia were the moisture levels were adequate [20]. The avian fossil representation for this stage is sparse and localized to Central and Western Europe, but suggests, though not significantly so, a higher degree of open landscape compared with any later period (Fig. 5). A spatial presentation of the forest bird species shows that they are primarily located south of the Alps (Fig. 7), in agreement with the palynological evidence. Among the most commonly found species in these areas are *Turdus viscivorus* (Mistle Thrush), *Turdus merula* (Eurasian Blackbird), *Garrulus glandarius* (Eurasian Jay), *Scolopax rusticola* (Eurasian Woodcock), *Columba palumbus* (Common Wood-pigeon) and *Otus scops* (Common Scops-owl), suggesting temperate mixed woodlands. However, finds of species like *Turdus iliacus* (Redwing) and *Aegolius funerous* (Boreal Owl) points toward boreal elements in Southern Europe as well. The Eastern European landscape cannot be deduced from the present results due to data scarcity, but a paleoecological study on avian fossils from the Bisnik Cave in Poland report *Tetrao tetrix, Corvus monedula* (Eurasian Jackdaw) and *Lagopus lagopus* [38], pointing towards the presence of open woodlands.

European interglacial vegetation structure has been much discussed (e.g. [21]). Pollen studies suggest that coniferous and deciduous forests dominated the vegetation during the warmer periods, with dwarf shrubs and herbaceous plants prevalent during colder intervals [17]–[19], [79], [80]. One third of the sites from MIS 5 date to the Eemian and are truly interglacial while the rest stem from subsequent stadials and interstadials within MIS 5. The results show that during the entire MIS 5 the Western Palearctic had significant higher proportion of forest-adapted bird species (Table S1, 28.5%) compared with later stages of colder character, somewhat in agreement with traditional views of a forest-covered interglacial landscape. The high proportion of open-adapted bird species (Table S1, 32.6%) for this stage, however, also suggests a substantial degree of openness, even so within the interglacial per se (Fig. 7). Species of temperate grasslands like *Crex crex, Perdix perdix* and *Coturnix coturnix* are frequent in the fossil record for the entire MIS 5, including the Eemian, along with the more cold-tolerant *Lagopus lagopus*, which was, however, only found from sites dated to the stadials and interstadials after MIS 5e. Northwestern Europe exhibited equally strong patterns regarding openness, despite one out of three sites being from the Eemian. *Crex crex, Perdix perdix* and *Coturnix coturnix* are also found in this region along with *Corvus corone* (Carrion Crow) *Carduelis carduelis* (European Goldfinch) and *Alauda arvensis* (Eurasian Skylark), all suggesting at least partially open landscapes. Throughout the Western Palearctic, these open-vegetation species could have survived in open areas maintained by grazing megafauna as well as marginal edaphic conditions, fluvial activity, windthrows and forest fires [21].

The Early and Middle Weichselian (MIS 3-4) trended towards colder temperatures and during the coldest periods, polar desert and steppe-tundra prevailed with tree species primarily found in Southern European refugia [20]. During warmer intervals shrubs and tree species expanded. Forest bird species are found south of a tilted line from the Pyrenees in the west across Central Europe just north of the Alps and in Eastern Europe as high as the 50°N latitude at Raj Cave in Poland (PO21) (Fig. 1: site 9 and Fig. 7). This spatial distribution corresponds well with the many findings of tree macrofossils in Eastern Europe [29], [30], reporting both boreal coniferous and temperate deciduous tree species from the region during the late MIS 3 when conditions were mildest. In Northwestern Europe, pollen studies indicates that the landscape was dominated by polar desert to the north and steppe-tundra to the south during MIS 4 while the warmer parts of MIS 3 had open woodland of *Picea* (Spruce), *Pinus* (Pine) and *Betula* (Birch) to the south and treeless shrub tundra to the north [18], [20], [81], something which is supported by mammalian fossil assemblages [72]. There have, however, been pollen findings of *Betula* sp., *Juniperus* sp. and *Pinus* sp. dated to early MIS 3 as far north as Denmark [82]. The avifaunal results presented here suggest a relatively open landscape in Northwestern Europe at this time with only a few occurrences of woodland species, notably *Turdus philomelos* (Song Trush) *Turdus viscivorus* and *Turdus merula*, all from central France (Fig. 6 and Fig. 7).

During the Last Glacial Maximum (MIS 2: LGM, 18,000–22,000 years BP), the Fennoscandian ice sheet reached as far south as the 50°N latitude in Poland and Germany [83], close to some of the sites dated to this period. Pollen evidence indicates a prevalence of tundra north of the Alps and steppe-like vegetation with few trees in lowland Southern Europe, with most trees mostly restricted to mountain belts of suitable conditions. However, findings of macrofossils of coniferous and deciduous tree species in Hungary (dated 32,500–16,500 years BP) [29], as well as conifer species in southern Poland and deciduous tree species in Austria (dated 30,000–25,000 years BP) [30], indicates a presence of trees further north. This is further supported by findings of *Pinus* sp., *Picea* sp., *Alnus* sp. (Alder), shrubby *Betula* sp. and *Salix* sp. (Willow) further north and east in Belarus and Ukraine [31] and findings of a continuous presence of forest species of birds and mammals in the aforementioned Bisnik Cave in Poland [38]. Additionally, a species distribution modeling study have found suitable LGM conditions for several boreal tree species close to the Fennoscandian ice-sheet in Eastern Europe [35] and a genetic study on *Picea abies* (Norway Spruce) suggested that this species survived the full-glacial in microenvironmentally favorable pockets in western Norway [84]. LGM occurrences in Northwestern Europe are poorly represented in the avian fossil record, making it impossible to infer landscape characteristics in this area during this period. For the whole of the Western Palearctic, the proportion of forest birds for this particular cold period was on average 11% (Table S1), but as high as >20% for some sites in Spain, Italy, Germany and Poland (Fig. 7). The location of forest birds in Eastern and Central Europe suggests that some forest tree species were able to survive the full-glacial climate in places north of the traditionally recognized southern refugia. The three sites closest to the Fennoscandian ice sheet (Zwergenloch (BRD156) in Germany and Mamutowa (PO13) and Oblazowa Cave (PO35) in Poland) are interesting in relation to the debate on glacial forests (Fig. 1: site 10–12), as they all had bird species from various forest types. Among these were *Tetrao urogallus, Aegolius funereus* and *Asio otus* (Long-eared Owl) which prefers mature coniferous forest with some degree of open ground or clearings. Species of mixed as well as broad-leaved deciduous forest and woodland, like *Dendrocopos medius* (Middle Spotted Woodpecker), *Turdus merula* and *Coccothraustes coccothraustes* (Hawfinch) are also reported. It has been suggested that boreal trees like *Pinus, Picea, Betula, Juniperus* (Juniper), *Salix* and *Larix* (Larch) predominated in patchy woodland stands in Central and Eastern Europe, while more thermophilous species like *Quercus, Corylus* (Hazel), *Ulmus* (Elm) and *Tilia* (Linden) may have survived in localized pockets of favorable mild and humid conditions [29], for example in north/south oriented river valleys [26], [31], and the mixture of fossilized birds of different forest types presented here is in accordance with this hypothesis.

In the late Weichselian (post LGM MIS 2, but before the Late-Glacial) forest birds are found throughout Europe including areas further northwest in continental Europe and southern England (Fig. 7). The high proportion of forest birds in Northwestern Europe is in contrast to the impression made by pollen studies that found largely non-arboreal vegetation species in this region [26]. Importantly, the forest-bird proportion in this subregion is largely made up by *Turdus* spp., which, despite forests being considered a habitat of major importance, are able to breed in areas outside this type of vegetation, for example in scrublands [56]. Two other species are also well represented namely *Tetrao urogallus* and *Garrulus glandarius*, which are found in southern England and Belgium. Their presence indicates that well-developed forests were present in this subregion at times during the period. Simulation studies have shown that boreal tree species could have been present in Northwestern Europe at the LGM [35], [36], while the climate would have permitted both boreal and especially temperate types at 14,000 and 10,000 years BP [36], the latter also well-documented by paleoecological studies [85], [86].

The Late-Glacial (MIS 1/2) is a short period relative to the other stages, that spans the relatively warm Bølling-Allerød interstadials as well as the cold Younger Dryas stadial and is thus a period of rapid changes in climate, the effect of which depended heavily on geographic location, even within Northwestern Europe [51]. Consequently, vegetation responses differed geographically [86], but were sometimes very rapid [85]. At the start of the Late-Glacial, the continuous permafrost in Northwestern Europe became more sporadic or disappeared [82] and plant fossil data show a development of dense vegetation, initially by a rise in *Artemisia*, followed by woodlands of varying density of *Betula* spp., *Salix* spp. and *Juniperus communis* [85], [86] and appearance of *Populus tremula* (Aspen) in northwest Germany [87]. At the transition to the Younger Dryas, forests became increasingly open, transgressing to heath and tundra landscapes in Northwestern Europe [86]. For the Late-Glacial overall, the forest bird proportion in Northwest Europe increased compared with MIS 2 with the presence of species such as *Dendrocopos major* (Great Spotted Woodpecker), *Scolopax rusticola* (Eurasian Woodcock) and *Coccothraustes coccothraustes*. *Turdus* spp. are still represented, but make up smaller parts of assemblages compared with MIS 2. At the same time, there was still a high proportion of open-adapted species in Northwestern Europe, in particular *Lagopus lagopus, Lagopus muta, Corvus monedula*, and *Perdix perdix*, suggesting that assemblages within this MIS category stems from both warm and cold periods. However, the temporal classification of the data hinders any close inspection of the species' presences in relation to specific interstadial or stadial periods.

### Methodological Considerations

Applying the uniformitarian principle to deduce past environments by utilizing modern analogues require important assumptions. Firstly, the climate and vegetation attributes of each species in the study were inferred from their present ranges, it was thus assumed that these ranges in broad terms reflect the bioclimatic spaces that the species tolerate. Secondly, one needs to assume that the species had the same climatic tolerances and vegetation requirements as today to use their presence in space and time to infer environmental conditions during past periods. Although some niche evolution may have occurred in some species we have no reason to suspect large, general changes over this time period. Furthermore, fossils in general are subject to collection and preservation biases of both temporal and spatial character [88]. Reflecting this, the dataset was predominated by fossils younger than 75,000 years (Table 1) and by fossils from mountain cave sites in Western Europe, especially France and northern Spain (Fig. 1). The latter biases the overall spatial distribution of the results towards Western Europe, although Eastern Europe did have some coverage as well. In addition, the dataset were predominated by large-bodied species (Corvidae appeared most frequently with 14.6% of the fossils belonging to this family, followed by Anatidae (10.6%), Tetraonidae (8.0%), Turdidae (6.5%), Phasanidae (6.4%) and Strigidae (6.4%)) supporting the general view that fossil records are taxonomically biased towards species with robust bones. From the paleoecological perspective of the present study, this should not affect the results much as it should not lead to spatiotemporal biases. Furthermore, the species used here represents a fairly even share of open and woodland vegetation types. For investigating the range shifts, a mixture of sedentary and migratory species with relatively restricted modern distributions was considered. While the past distribution of sedentary species can be reliably inferred from fossils, the shifts of migrating species should be inferred with caution. This is especially evident with species like *Plectrophenax nivalis* (Fig. 2) that have fossil specimens in close proximity to present winter ranges, but also for sedentary species with irruptive behavior that presently are observed as vagrants in areas far away from native ranges (e.g. *Pinicola enucleator* and *Surnia ulula* [56] (Fig. 2)). Fossils of these species could therefore be remnants of vagrant individuals, although it seems unlikely that such vagrants could dominate the observed patterns.

### Conclusion

The avian fossil record from the latest Middle Pleistocene to the Late Pleistocene/Holocene transition documents sometimes strong range shifts in response to past climate change, linked to the climatic shifts per se, but even more so to vegetation changes. Present-day northern species of different vegetation types moved southwards, while open-vegetation species of the south and east moved north and westwards, respectively, during cold stages, the latter presumably due to increased aridity and openness. These conditions also favored alpine species causing them to shifts downwards into the European lowlands. The direct link to climate was primarily clear in Northwestern Europe, probably reflecting the particularly disruptive glacial climatic effects in this part of the Western Palearctic region. The close association between many bird species and vegetation types makes them useful for inferring woodland characteristics of past landscapes. Interestingly, the presence of forest birds in areas outside Southern Europe during the coldest stages, especially in East and Central Europe, are in agreement with the increasingly supported presence of trees in northern glacial refugia. In addition, the avifaunal record also suggests forest-dominated, but partially open landscapes during the Last Interglacial.

## Supporting Information

Figure S1 **Boxplots of vegetation and temperature properties for birds in the AvGenus supplementary analysis of the Western Palearctic (WP).** Boxplots shows the percentage of fossil bird species preferring cold-medium (Temp. B), medium-warm (Temp. D) and warm (Temp. E) conditions (Left) and open, mixed and forest vegetation (Right) in the Western Palearctic, for each MIS. Boxes show the median, 25th and 75th percentile and whiskers extending 1.5 interquartile range (IQR). Dot symbols identify outliers. Letters indicate significant relationships according to Wilcoxon signed-rank tests (p≤0.05). Proportions are overall similar to the main analysis, especially regarding the Forest variable.
(TIF)

Figure S2 **Boxplots of vegetation and temperature properties for birds in the AvGenus supplementary analysis of the Northwestern European subregion (NW).** Boxplots shows the percentage of fossil bird species preferring cold-medium (Temp. B), medium-warm (Temp. D) and warm (Temp. E) conditions (Left) and open, mixed and forest vegetation (Right) in the Western Palearctic, for each MIS. Boxes show the median, 25th and 75th percentile and whiskers extending 1.5 interquartile range (IQR). Dot symbols identify outliers. Letters indicate significant relationships according to Wilcoxon signed-rank tests (p≤0.05). The proportions of the vegetation variables are similar to the main analysis while the proportions for the temperature variables are less consistent.
(TIF)

Figure S3 **Boxplots of vegetation and temperature properties for birds in the NoPas supplementary analysis of the Western Palearctic (WP).** Boxplots shows the percentage of fossil bird species preferring cold-medium (Temp. B), medium-warm (Temp. D) and warm (Temp. E) conditions (Left) and open, mixed and forest vegetation (Right) in the Western Palearctic, for each MIS. Boxes show the median, 25th and 75th percentile and whiskers extending 1.5 interquartile range (IQR). Dot symbols identify outliers. Letters indicate significant relationships according to Wilcoxon signed-rank tests (p≤0.05). The proportions are similar to the main analysis. Forest is the only significant variable, with MIS 2 having significantly lower proportions than MIS 1/2, 5 and 6.
(TIF)

Figure S4 **Boxplots of vegetation and temperature properties for birds in the NoPas supplementary analysis of the Northwestern European subregion (NW).** Boxplots shows the percentage of fossil bird species preferring cold-medium (Temp. B), medium-warm (Temp. D) and warm (Temp. E) conditions (Left) and open, mixed and forest vegetation (Right) in the Western Palearctic, for each MIS. Boxes show the median, 25th and 75th percentile and whiskers extending 1.5 interquartile range (IQR). Dot symbols identify outliers. Letters indicate significant relationships according to Wilcoxon signed-rank tests (p≤0.05). The proportions of both temperature and vegetation variables are consistent with the main analysis.
(TIF)

Table S1 **Average proportion of birds for each climate and vegetation category.** Proportions for cold stages consist of averaged data from MIS 2, 3–4 and 6 (though MIS 6 is not included in proportions for Northwestern Europe), while proportions for warm stage consist of data from MIS 5 only. Proportions for LGM consists of data from MIS 2 that are dated between 22–18 kya BP. The remainder of the habitat categories (Marine, Wetlands, Rocky and Aerial) included in the database are not treated in this study, hence Open, Mixed and Forest does not amount to 100%.
(DOCX)

Table S2 **Results of Kruskal–Wallis one-way analysis of variance for the AvGenus supplementary analyses.** AvGenus  =  Average genus. In these analyses the habitat and climate attributes of each bird species were replaced with the genus average providing a conservative estimate of the potential impact of uncertainty in species-level fossil identification, most of which pertain species within the same genus.
(DOCX)

Table S3 **Results of Kruskal–Wallis one-way analysis of variance for the NoPas supplementary analyses.** NoPas  =  No Passerines. These analyses excluded Passerines, which are often perceived as a group with problematic species-specific fossil identification.
(DOCX)

Dataset S1 **Dataset of fossilized birds in Europe used in this study.** Based on Tyrberg (1998, 2008).
(XLSX)
