# Supplementary figures and images for: 180,000 Years of Climate Change in Europe: Avifaunal Responses and Vegetation Implications
Source: PLoS One. 2014 Apr 9;9(4):e94021. doi: 10.1371/journal.pone.0094021 (PMC3981757; doi:10.1371/journal.pone.0094021)

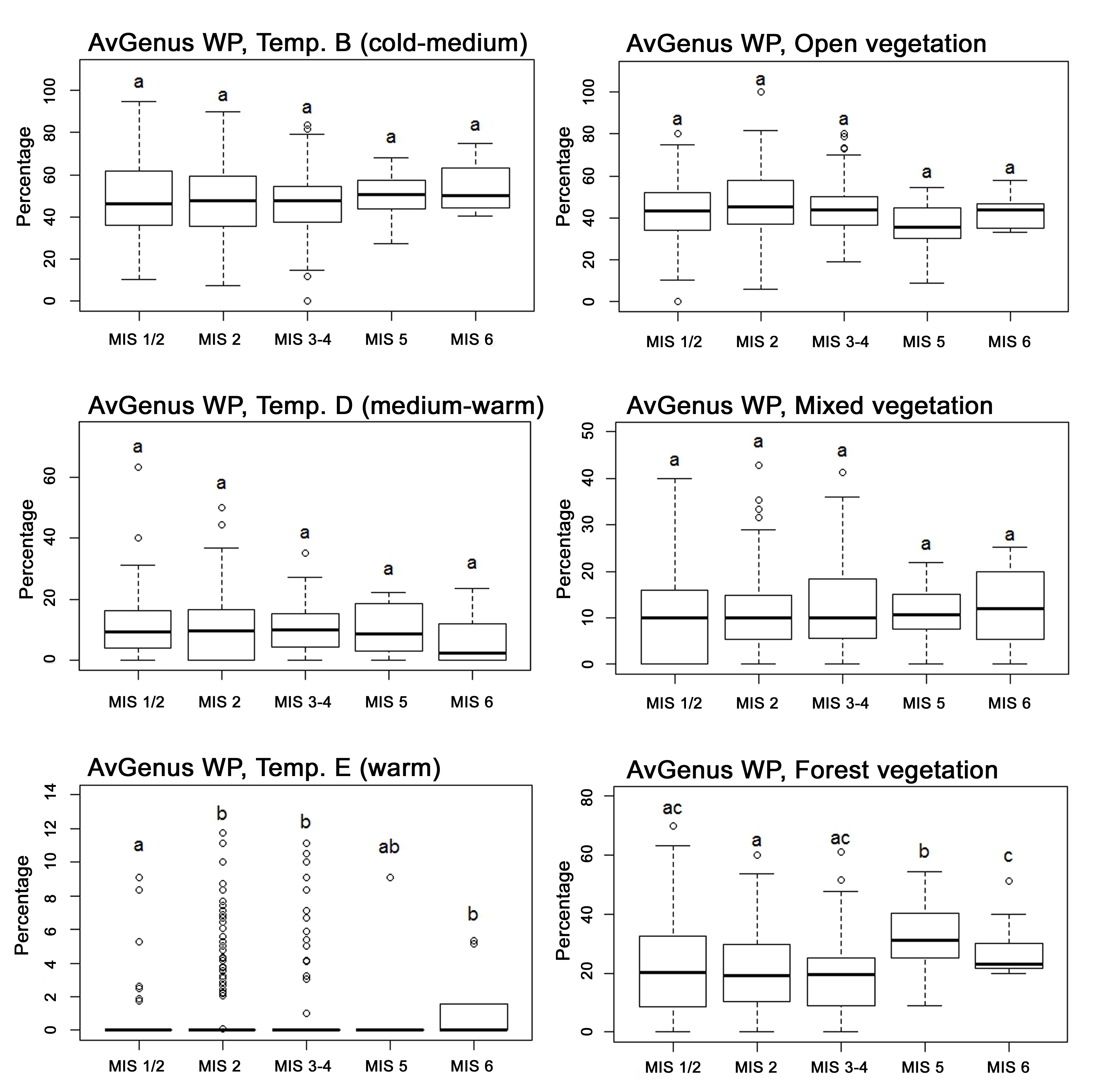

Supplement: Figure S1 — Boxplots of vegetation and temperature properties for birds in the AvGenus supplementary analysis of the Western Palearctic (WP). Boxplots shows the percentage of fossil bird species preferring cold-medium (Temp. B), medium-warm (Temp. D) and warm (Temp. E) conditions (Left) and open, mixed and forest vegetation (Right) in the Western Palearctic, for each MIS. Boxes show the median, 25th and 75th percentile and whiskers extending 1.5 interquartile range (IQR). Dot symbols identify outliers. Letters indicate significant relationships according to Wilcoxon signed-rank tests (p≤0.05). Proportions are overall similar to the main analysis, especially regarding the Forest variable. (TIF) [file pone.0094021.s001.tif]

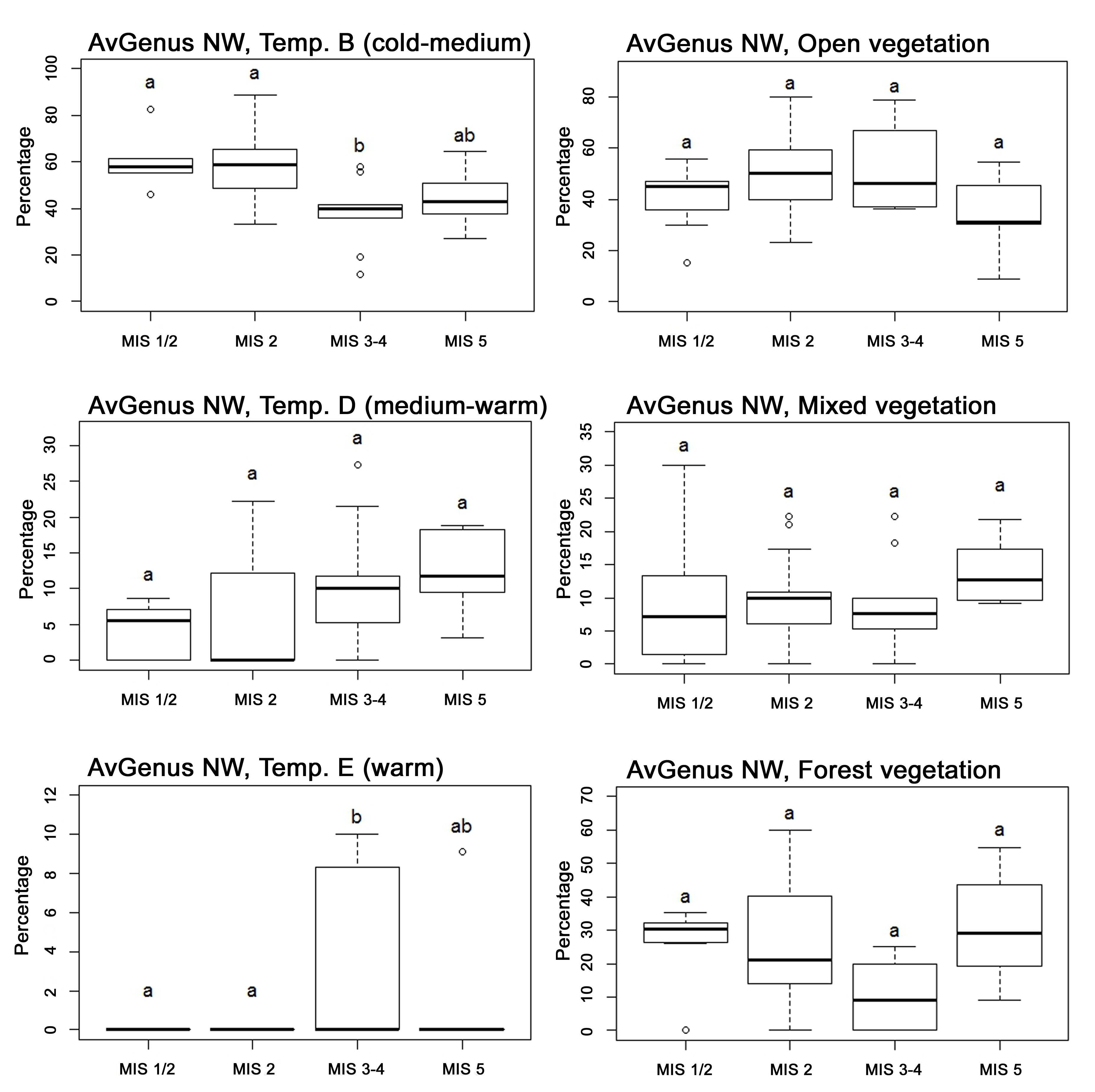

Supplement: Figure S2 — Boxplots of vegetation and temperature properties for birds in the AvGenus supplementary analysis of the Northwestern European subregion (NW). Boxplots shows the percentage of fossil bird species preferring cold-medium (Temp. B), medium-warm (Temp. D) and warm (Temp. E) conditions (Left) and open, mixed and forest vegetation (Right) in the Western Palearctic, for each MIS. Boxes show the median, 25th and 75th percentile and whiskers extending 1.5 interquartile range (IQR). Dot symbols identify outliers. Letters indicate significant relationships according to Wilcoxon signed-rank tests (p≤0.05). The proportions of the vegetation variables are similar to the main analysis while the proportions for the temperature variables are less consistent. (TIF) [file pone.0094021.s002.tif]

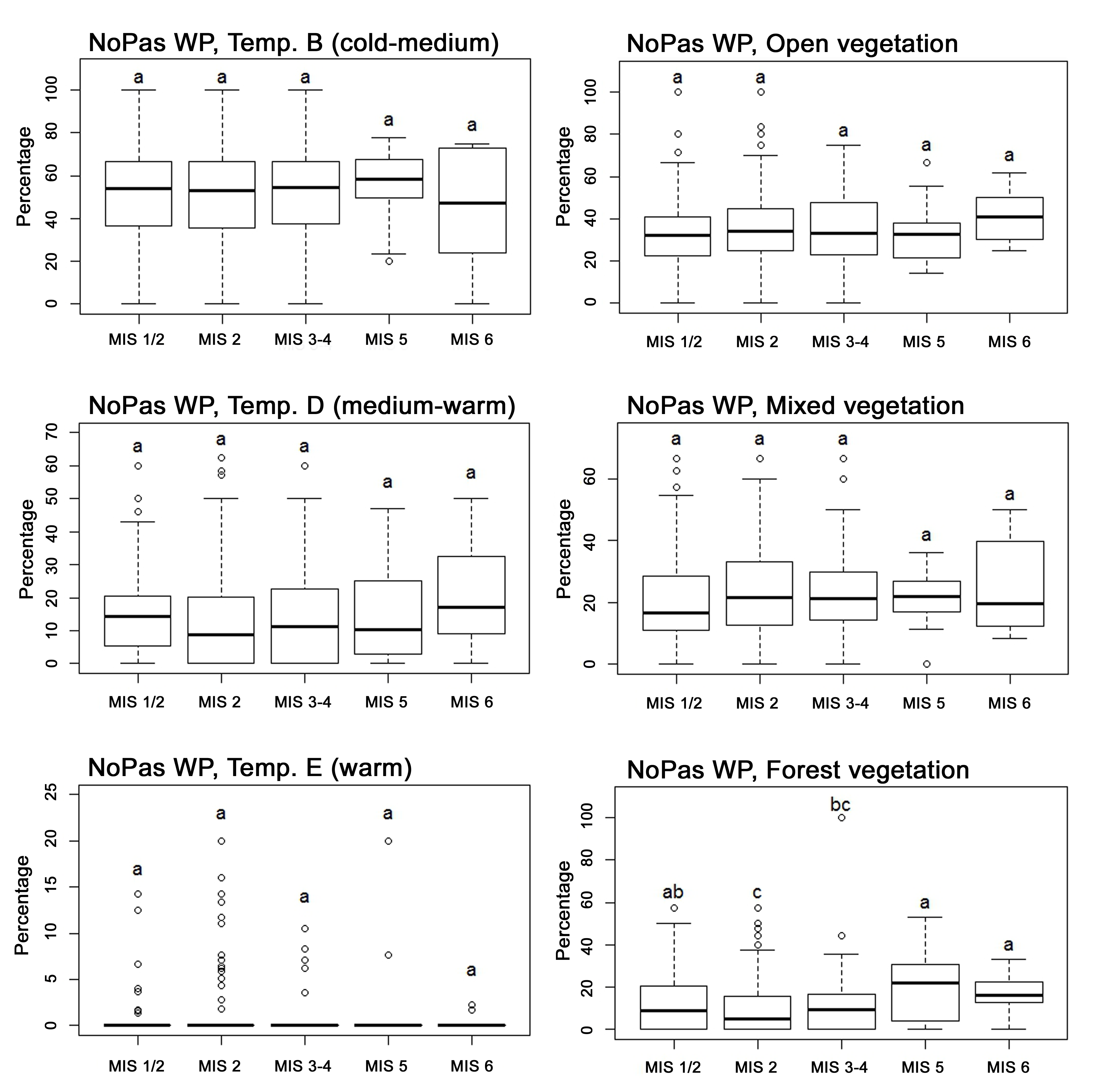

Supplement: Figure S3 — Boxplots of vegetation and temperature properties for birds in the NoPas supplementary analysis of the Western Palearctic (WP). Boxplots shows the percentage of fossil bird species preferring cold-medium (Temp. B), medium-warm (Temp. D) and warm (Temp. E) conditions (Left) and open, mixed and forest vegetation (Right) in the Western Palearctic, for each MIS. Boxes show the median, 25th and 75th percentile and whiskers extending 1.5 interquartile range (IQR). Dot symbols identify outliers. Letters indicate significant relationships according to Wilcoxon signed-rank tests (p≤0.05). The proportions are similar to the main analysis. Forest is the only significant variable, with MIS 2 having significantly lower proportions than MIS 1/2, 5 and 6. (TIF) [file pone.0094021.s003.tif]

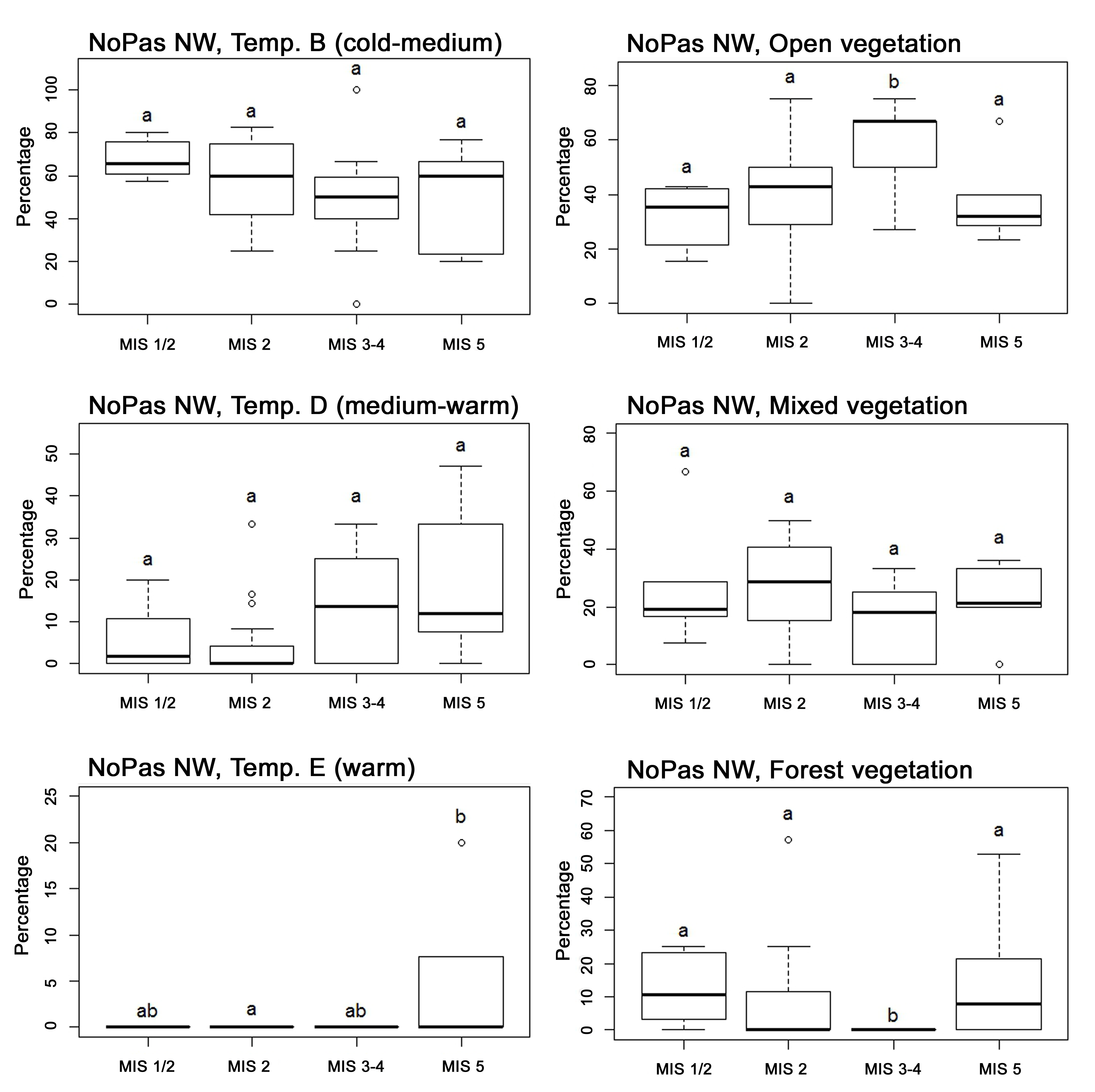

Supplement: Figure S4 — Boxplots of vegetation and temperature properties for birds in the NoPas supplementary analysis of the Northwestern European subregion (NW). Boxplots shows the percentage of fossil bird species preferring cold-medium (Temp. B), medium-warm (Temp. D) and warm (Temp. E) conditions (Left) and open, mixed and forest vegetation (Right) in the Western Palearctic, for each MIS. Boxes show the median, 25th and 75th percentile and whiskers extending 1.5 interquartile range (IQR). Dot symbols identify outliers. Letters indicate significant relationships according to Wilcoxon signed-rank tests (p≤0.05). The proportions of both temperature and vegetation variables are consistent with the main analysis. (TIF) [file pone.0094021.s004.tif]
