# Supplementary material for: 180,000 Years of Climate Change in Europe: Avifaunal Responses and Vegetation Implications
Source: PLoS One. 2014 Apr 9;9(4):e94021. doi: 10.1371/journal.pone.0094021 (PMC3981757; doi:10.1371/journal.pone.0094021)
Supplement: Table S1 — Average proportion of birds for each climate and vegetation category. Proportions for cold stages consist of averaged data from MIS 2, 3–4 and 6 (though MIS 6 is not included in proportions for Northwestern Europe), while proportions for warm stage consist of data from MIS 5 only. Proportions for LGM consists of data from MIS 2 that are dated between 22–18 kya BP. The remainder of the habitat categories (Marine, Wetlands, Rocky and Aerial) included in the database are not treated in this study, hence Open, Mixed and Forest does not amount to 100%. (DOCX) [file pone.0094021.s005.docx]

Table S1: Average proportion of birds for each climate and vegetation category.

|  | **Western Palearctic** | | | | **Northwestern Europe** | | |
| --- | --- | --- | --- | --- | --- | --- | --- |
|  | Average (%) | Cold stages (%) | Warm stage (%) | LGM (%) | Average (%) | Cold stages (%) | Warm stage (%) |
| Temperature A | 4.64 | 4.16 | 5.43 | 5.32 | 7.68 | 7.70 | 7.48 |
| Temperature B | 47.61 | 46.41 | 50.78 | 35.12 | 48.55 | 43.86 | 44.70 |
| Temperature C | 35.78 | 37.33 | 31.28 | 42.26 | 33.31 | 37.30 | 31.84 |
| Temperature D | 11.11 | 11.27 | 11.12 | 15.84 | 9.18 | 10.23 | 12.63 |
| Temperature E | 0.86 | 0.80 | 1.40 | 1.46 | 1.29 | 0.90 | 3.36 |
| Humidity A | 6.57 | 5.96 | 8.18 | 7.11 | 3.38 | 3.36 | 1.40 |
| Humidity B | 38.34 | 38.73 | 39.99 | 40.43 | 38.58 | 38.66 | 41.29 |
| Humidity C | 31.43 | 32.34 | 28.60 | 25.08 | 30.07 | 27.22 | 29.81 |
| Humidity D | 19.52 | 18.43 | 20.01 | 20.78 | 22.11 | 23.78 | 22.07 |
| Humidity E | 4.14 | 4.51 | 3.23 | 6.60 | 5.86 | 6.97 | 5.43 |
| Open | 38.91 | 41.81 | 32.63 | 45.29 | 40.19 | 45.03 | 37.20 |
| Mixed | 17.87 | 18.33 | 16.25 | 21.60 | 19.18 | 20.39 | 14.45 |
| Forest | 20.33 | 18.02 | 28.49 | 11.28 | 18.14 | 12.94 | 26.45 |

Proportions for cold stages consist of averaged data from MIS 2, 3-4 and 6 (though MIS 6 is not included in proportions for Northwestern Europe), while proportions for warm stage consist of data from MIS 5 only. Proportions for LGM consists of data from MIS 2 that are dated between 22-18 kya BP. The remainder of the habitat categories (Marine, Wetlands, Rocky and Aerial) included in the database are not treated in this study, hence Open, Mixed and Forest does not amount to 100%.
