# Supplementary material for: 180,000 Years of Climate Change in Europe: Avifaunal Responses and Vegetation Implications
Source: PLoS One. 2014 Apr 9;9(4):e94021. doi: 10.1371/journal.pone.0094021 (PMC3981757; doi:10.1371/journal.pone.0094021)
Supplement: Table S2 — Results of Kruskal–Wallis one-way analysis of variance for the AvGenus supplementary analyses. AvGenus = Average genus. In these analyses the habitat and climate attributes of each bird species were replaced with the genus average providing a conservative estimate of the potential impact of uncertainty in species-level fossil identification, most of which pertain species within the same genus. (DOCX) [file pone.0094021.s006.docx]

Table S2: Results of Kruskal–Wallis one-way analysis of variance for the AvGenus supplementary analyses.

|  | **Western Palearctic** | | | **Northwestern Europe** | | |
| --- | --- | --- | --- | --- | --- | --- |
| **Variable** | ***χ*^2^** | **Df** | **p** | ***χ*^2^** | **Df** | **p** |
| Temperature A | 3.7846 | 4 | 0.4359 | 0.4118 | 3 | 0.9378 |
| Temperature B | 3.3551 | 4 | 0.5003 | 10.7001 | 3 | **0.01346** |
| Temperature C | 8.1516 | 4 | 0.08618 | 6.1627 | 3 | 0.104 |
| Temperature D | 2.3262 | 4 | 0.0676 | 6.1861 | 3 | 0.1029 |
| Temperature E | 13.2271 | 4 | **0.01022** | 12.2939 | 3 | **0.006441** |
| Humidity A | 7.0489 | 4 | 0.1333 | 9.0768 | 3 | **0.02829** |
| Humidity B | 5.6079 | 4 | 0.2304 | 4.6867 | 3 | 0.1962 |
| Humidity C | 2.3921 | 4 | 0.6641 | 3.4912 | 3 | 0.3219 |
| Humidity D | 11.0454 | 4 | **0.02606** | 6.8999 | 3 | 0.07516 |
| Humidity E | 11.8144 | 4 | **0.01879** | 4.7528 | 3 | 0.1908 |
| Vegetation Open | 9.427 | 4 | 0.05127 | 4.5401 | 3 | 0.2087 |
| Vegetation Mixed | 1.5977 | 4 | 0.8092 | 3.4193 | 3 | 0.3314 |
| Vegetation Forest | 11.9131 | 4 | **0.01801** | 7.5923 | 3 | 0.05523 |

AvGenus = Average genus. In these analyses the habitat and climate attributes of each bird species were replaced with the genus average providing a conservative estimate of the potential impact of uncertainty in species-level fossil identification, most of which pertain species within the same genus.
