# Supplementary material for: 180,000 Years of Climate Change in Europe: Avifaunal Responses and Vegetation Implications
Source: PLoS One. 2014 Apr 9;9(4):e94021. doi: 10.1371/journal.pone.0094021 (PMC3981757; doi:10.1371/journal.pone.0094021)
Supplement: Table S3 — Results of Kruskal–Wallis one-way analysis of variance for the NoPas supplementary analyses. NoPas = No Passerines. These analyses excluded Passerines, which are often perceived as a group with problematic species-specific fossil identification. (DOCX) [file pone.0094021.s007.docx]

Table S3: Results of Kruskal–Wallis one-way analysis of variance for the NoPas supplementary analyses.

|  | **Western Palearctic** | | | **Northwestern Europe** | | |
| --- | --- | --- | --- | --- | --- | --- |
| **Variable** | ***χ*^2^** | **Df** | **p** | ***χ*^2^** | **Df** | **p** |
| Temperature A | 0.436 | 4 | 0.9794 | 2.1119 | 3 | 0.5495 |
| Temperature B | 1.0703 | 4 | 0.8989 | 3.9007 | 3 | 0.2724 |
| Temperature C | 8.2528 | 4 | 0.08274 | 6.332 | 3 | 0.09653 |
| Temperature D | 7.9309 | 4 | 0.09414 | 5.9215 | 3 | 0.1155 |
| Temperature E | 4.5638 | 4 | 0.3351 | 14.76 | 3 | **0.002034** |
| Humidity A | 4.8 | 4 | 0.3084 | 4.0471 | 3 | 0.2564 |
| Humidity B | 5.4954 | 4 | 0.2401 | 4.2898 | 3 | 0.2318 |
| Humidity C | 0.7979 | 4 | 0.9387 | 0.8037 | 3 | 0.8486 |
| Humidity D | 2.1639 | 4 | 0.7057 | 1.9309 | 3 | 0.5869 |
| Humidity E | 6.8734 | 4 | 0.1427 | - | 3 | - |
| Vegetation Open | 3.7121 | 4 | 0.4464 | 9.199 | 3 | **0.02676** |
| Vegetation Mixed | 5.1216 | 4 | 0.275 | 3.2939 | 3 | 0.3485 |
| Vegetation Forest | 16.0543 | 4 | **0.002947** | 9.3072 | 3 | **0.02547** |

NoPas = No Passerines. These analyses excluded Passerines, which are often perceived as a group with problematic species-specific fossil identification.
